# Multiple Ring Electrode-Based PMUT with Tunable Deflections

**DOI:** 10.3390/mi16060623

**Published:** 2025-05-25

**Authors:** Jan Helmerich, Manfred Wich, Annika Hofmann, Thomas Schaechtle, Stefan Johann Rupitsch

**Affiliations:** 1 Laboratory for Electrical Instrumentation and Embedded Systems, Department of Microsystems Engineering (IMTEK), University of Freiburg, 79110 Freiburg, Germany; 2 BrainLinks-BrainTools, 79110 Freiburg im Breisgau, Germany

**Keywords:** adjustable deflection, characterization of piezoelectric materials, multiple electrode design, PMUT, PZT annealing, ultrasonic transducers

## Abstract

Ultrasonic applications such as non-destructive testing, biomedical imaging or range measurements are currently based on piezoelectric bulk transducers. Yet, these kinds of transducers with their mm to cm dimensions are rather impractical in fields in which both frequencies in the kHz region and small-feature sizes are required. This fact mainly relates to the inverse relationship between the resonance frequency constant and the transducers’ dimension, yielding a higher frequency and attenuation with a decreasing size. Piezoelectric micromachined ultrasonic transducers (PMUTs), in comparison, incorporate a small-scale µm design while preserving the operating frequency in the desired kHz range. This contribution presents the detailed manufacturing of such a PMUT with a multiple ring electrode-based structure to additionally adjust the sound pressure fields. The PMUT will be characterized by its deflection in air along with the characterization of the piezoelectric material lead zirconate titanate (PZT) itself. The measurements showed a maximum polarization of 21.8 µC/cm^2^ at 50 kV/cm, the PMUT’s displacement of 30.50 nm/V in air when all electrodes are driven, and an adjustable deflection via different electrode excitations without the need for additional hardware. The ring design also offered the possibility to emit two distinct frequencies simultaneously. These results demonstrate the potential of the designs for small-feature-size applications as they are in high demand for implantable devices, miniaturized ultrasonic-based communication or drug delivery.

## 1. Introduction

Ultrasonic applications such as non-destructive testing, ultrasonic imaging, ultrasonic-based communication, and drug delivery heavily rely on the use of bulk transducers [1,2,3,4,5,6]. However, the desire to compact devices in specific fields of ultrasonic-based implementations demands the miniaturization of both the electronic components and the ultrasonic transducer. Related ultrasonic-based technologies include fingerprint sensors, particle manipulation, acoustic power transmission, and ultrasonic-based communication for implantable devices [7,8]. Due to the inverse relationship between geometrical dimensions and resonance frequencies, designs founded on piezoelectric discs tend to feature increasing resonance frequencies with a decreasing diameter or thickness. This fact, in turn, results in an increasing damping of the emitted ultrasonic wave due to the surrounding medium.

Micromachined ultrasonic transducers (MUTs), in contrast, maintain the frequency in the kHz and lower MHz range for which the medium’s damping will hardly affect the functionality of the overall system. Depending on the underlying concept, these MUTs are categorized as capacitive ultrasonic micromachined transducers (CMUTs) or piezoelectric micromachined ultrasonic transducers (PMUTs), utilizing either the electrostatic attraction and repulsion, or the direct and inverse piezoelectric effect for transmitting and receiving ultrasonic waves, respectively. Both MUT principles are based on a diaphragm often referred to as a membrane and, therefore, allow for an intrinsic acoustic impedance matching. The MUT’s acoustic impedance Z can be approximated by

(1) $$Z=\underset{Radiation\;Impedance}{\underbrace{\frac{Z_{0}}{\cos\varphi }}}+\underset{Reactive\;Impedance}{\underbrace{\frac{s}{j2\pi f\;}},}$$

whereas $Z_{0}$ denotes the surrounding medium’s acoustic impedance, φ the radiation angle, s the stiffness of the vibrating diaphragm, f the excitation frequency and j the imaginary unit associated with the reactive part of the impedance [9]. Equation (1) yields an almost perfectly matched acoustic impedance to the surrounding medium for perpendicular radiation to the deflecting layer and an increasing excitation frequency.

For their proper operation, CMUTs require high DC bias voltages, often ranging between 30 V and 100 V [7,10,11,12,13]. These DC bias voltages can either be applied constantly via an external DC bias or via self-biasing circuits. The latter method provides the required high DC voltages from incident waves in combination with a voltage multiplier and rectification circuitry powered by a regular battery source, similar to a boost-up converter [11]. Alternatively, CMUTs can be pre-charged, i.e., an internal trapping layer is charged to provide an intrinsic DC voltage for operation [10]. Yet, these DC bias voltages or trapped charges pose a potential safety hazard for wearable or implantable devices. In contrast, PMUTs do not require such configurations for efficient operation. Additionally, PMUTs are less susceptible to parasitic effects as they do not rely on changes in capacitance to detect ultrasonic waves, feature a linear response, and enable high output pressures [13,14]. It is for these reasons that the following will solely focus on PMUTs.

**Figure 1:**
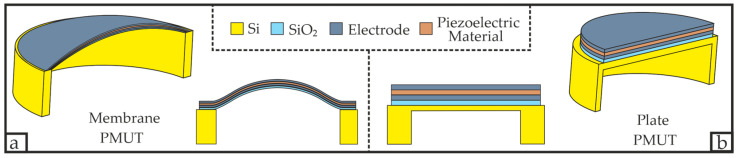
Comparison between a membrane PMUT (m-PMUT) and a plate PMUT (p-PMUT): (**a**) membrane PMUT with its intrinsic stress causing it to deform without an electric field; (**b**) plate PMUT with a thicker diaphragm and, therefore, dominated by the flexural rigidity *D*.

PMUTs, themselves, are classified into two subcategories referred to as plate PMUTs (p-PMUTs) and membrane PMUTs (m-PMUTs) [15]. m-PMUTs are pretension dominated, i.e., an intrinsic mechanical stress of the membrane is related to the existence of a net pretension T in the layers and causes the layers to already deform without the presence of an electric field (see Figure 1a). p-PMUTs, in turn, are dominated by the flexural rigidity D, i.e., their layers are relatively thick, their neutral axis is located in the device layer and their fundamental vibration modes can be derived by the clamped plate theory (see Figure 1b) [8].

In the study by Wah, the author derived the clamped plate theory and its resonance frequencies, and also introduced the differentiation between the analytical analysis of membrane-like and plate-like behavior [16]. In this contribution, the plate theory approaches the natural frequencies of a membrane when the non-dimensional parameter α, determined by a Bessel-based equation, approaches the roots μ of the Bessel function of the first kind $J_{n}(\mu ).$ Additionally, the condition

(2) $$\frac{\alpha^{2}D}{2r^{2}T}<<1$$

needs to hold, where r denotes the plate’s radius. Independently of the categorization as m-PMUT or p-PMUT, the application of an AC voltage results in the vibration of the PMUT due to the inverse piezoelectric effect and, thus, the emission of ultrasonic waves. If ultrasonic waves are detected, the direct piezoelectric effect enables the generation of charges at the piezoelectric layer, which in turn, allows for the measuring of the incident wave.

The very first working PMUT originates back to 1983, in which Royer et al. released a sputtered zinc oxide (ZnO) diaphragm and achieved a sensitivity of 25 µV/µbar [17]. Seven years later, Udayakumar et al. introduced the fabrication of sol–gel lead zirconate titanate (PZT) and demonstrated its potential to drive micromotors [18,19]. This sol–gel deposition technique, referred to as chemical solution deposition (CSD), became one of today’s standard approach for PZT-based PMUTs. Due to the lead content and the desire for better complementary metal-oxide semiconductor (CMOS) incorporation, alternative materials have been investigated, with Shelton et al. probably being the first to use an aluminum nitride (AlN)-based piezoelectric layer [20]. Wang et al. demonstrated the use of scandium-doped AlN as an active layer, with its potential to show three times greater transmitting performance than AlN-fabricated PMUTs manufactured for the same publication [21]. Despite these results, PZT remains to date the material of choice for actuators due to its superior piezoelectric properties for transmitting ultrasonic waves [1]. In order to achieve these properties, ferroelectric materials such as PZT need to be polarized first as they exhibit spontaneous polarization. This polarization process describes the alignment of dielectric poles in a single direction under the influence of an applied electric field [1].

Besides different materials, alternations of PMUTs’ shapes have also been investigated and include island-shaped, pre-concaved, rectangular-shaped and tent-plate-piston-shaped structures [22,23,24,25]. Additionally, a multiple top-electrode design has been suggested by Sammoura et al. in [26] and Liu et al. in [27], for which a theoretical model has been derived in each publication. In both [14,28], a two-electrode PMUT design was investigated. However, as of now and as far as we are aware, such a multiple-electrode design is still not state of the art for PMUTs, which allows each of the multiple electrodes to be driven individually. Still, the multiple ring structure suggests several advantages such as better receiving sensitivity of incident waves [17]. This aspect relates to the fact that for proper poling of the piezoelectric material, the inner part of the PMUT is subject to tension, whereas the outer part is compressed. This results in different signs of the detected signal and, therefore, the ability to detect low-intensity ultrasonic waves. Additionally, several electrodes provide redundancy as each electrode can potentially deflect the transducer. This proves to be promising for devices, in which a non-functional electrode results in a complete device malfunction. Therefore, the redundancy ensures that the PMUT remains operational even if one electrode fails.

It is for this reason that our contribution details the fabrication of such a ring-structured PMUT with an additional third electrode, in contrast to [14,28], which only investigated two electrodes covering the transducer. The second section describes the fabrication process of the PMUT and the material characterization of the PZT, including the P–E hysteresis loop and I–V curve. Instead of employing the state-of-the-art chemical solution deposition (CSD), we deposit the material PZT via sputtering. We also provide a detailed description of critical parameters and considerations in terms of sputtering PZT in order to clarify the process choices. Moreover, we describe the annealing process in depth along with the actually measured temperature profile, which is often only partially included in reports. The third section addresses the PMUT’s resonance frequency and deflection in air via a laser Doppler vibrometer (LDV). This investigation examines the capability of each electrode to deflect the PMUT’s membrane, and the combined use of two electrodes to emit two distinct frequencies simultaneously.

## 2. Device Fabrication and Material Characterization

**Figure 2:**
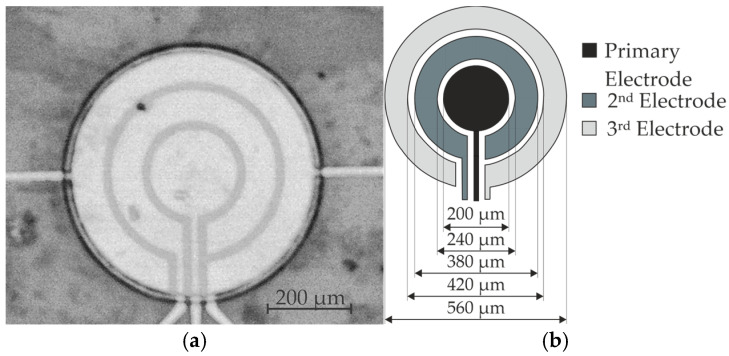
Fabricated PMUT and its schematic: (**a**) fabricated PMUT; (**b**) schematic of the multiple ring structure and their respective diameters.

The fabricated PMUT, along with its schematic can be seen in Figure 2. The primary electrode covers approximately an area of $A_{1}$ = 3.14 × 10^4^ µm^2^, the 2nd electrode covers $A_{2}$ = 6.8 × 10^4^ µm^2^ and the 3rd electrode features a footprint of $A_{3}$ = 1.2 × 10^5^ µm^2^. The electrode radii were chosen on the requirement that each electrode should be able to induce a membrane deflection, whereas the spacing of 20 µm between the electrodes was constrained by the resolution of the photolithography process.

### 2.1. PMUT Fabrication Process

The fabrication of the multiple ring-structured PMUTs is based on a 4-inch (100) Si wafer with a thickness of 300 µm. For the patterning of all structures, we chose to use the negative resist ma-N1440 (micro resist technology, Berlin, Germany) due to it withstanding higher temperatures of up to 160 °C. Therefore, the resist is suitable for both sputtering and deep silicon etching (DSE) [29]. Before each deposition, each target is pre-sputtered for at least 3 min to minimize impurities.

**Figure 3:**
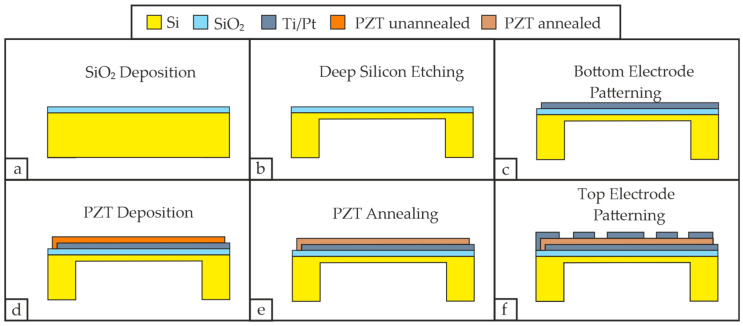
PMUT fabrication process. (**a**) SiO_2_ deposition via reactive sputtering; (**b**) deep silicon etching, creating cavities; (**c**) bottom electrode patterning and deposition; (**d**) PZT patterning, and deposition via sputtering; (**e**) annealing of the piezoelectric layer, inducing the change from the amorphous to the perovskite phase; (**f**) patterning and deposition of top electrode.

The first step involves the deposition of 300 nm SiO_2_ via reactive sputtering as seen in Figure 3a. This layer provides electrical insulation between the bottom and top electrode but also functions as a diffusion barrier layer, i.e., preventing the diffusion of Si to the PZT layer and the PZT’s oxygen O diffusion to the Si substrate [30]. Consequently, it also reduces the annealing temperature for PZT [30].

The required cavity is then created by a Versaline-PlasmaTherm DSE (deep silicone etching) process aimed at 295 µm, releasing the membrane as seen in Figure 3b.

The following process involves the patterning of the bottom electrode, for which 10 nm Ti is deposited, followed by 170 nm of Pt (see Figure 3c). The vacuum persisted during the change in the deposited material, i.e., the Ti was not exposed to oxygen before the Pt electrode was formed. The Pt electrode itself serves as a lattice template for the PZT and features a maximum lattice-constant mismatch of 3% in case of c-axis-oriented PZT [31]. The Ti layer, in comparison, functions as the adhesion layer and is also beneficial for promoting the crystallization of PZT due to the tunneling of Ti atoms [32]. Even though we do not sputter Ti and Pt simultaneously, we kept the Ti/Pt ratio at 5.8% for two reasons. First of all, if there is a deficiency in Ti atoms, PZT might tend to form the pyrochlore phase [32]. Secondly, if the Ti content exceeds a certain ratio, PZT may form lead oxides [32]. In both cases, PZT may not feature the perovskite phase after the annealing process. The sputter process of Figure 3c is conducted at an ambient temperature as suggested in [33] since this may support the formation of 100 textured perovskite PZT.

Growing the 1 µm PZT layer is conducted by sputtering a Pb(Zr_0.52_Ti_0.48_)O_3_-target at ambient temperature as well (see Figure 3d), and this is chosen over the CSD method due to a stronger interface connection to the bottom electrode, less impurity and better crystallinity [34].

**Figure 4:**
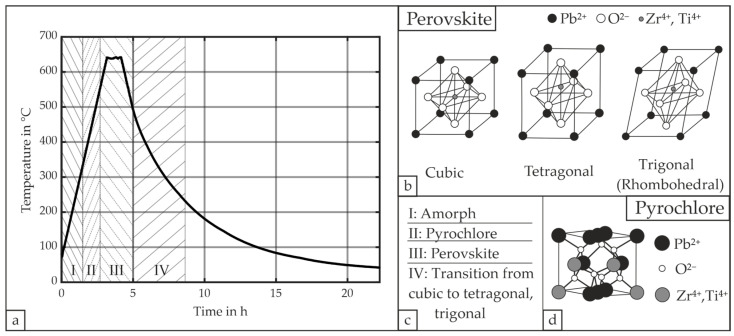
Target process temperature and the annealing procedure of PZT: (**a**) temperature profile for the annealing process with different critical stages I–IV of the annealing process; (**b**) PZT in its perovskite phase (PbTiO_3_, PbZrO_3_), with its cubic, tetragonal or trigonal symmetry; (**c**) different stages denoting the annealing process and the material’s symmetry, with (I) referring to the amorphous phase, (II) to the pyrochlore phase, (III) to the mostly perovskite phase with a co-existence of a pyrochlore phase, and (IV) the transition of the PZT in its perovskite phase from the cubic symmetry to either the tetragonal or trigonal symmetry; (**d**) PZT in its pyrochlore phase (Pb_2_Ti_2_O_6_O’, Pb_2_Zr_2_O_6_O’) according to [39,40].

The thin-film deposition is proceeded by an air-annealing process to induce the PZT’s transformation into the perovskite phase (see Figure 3e). This annealing process is performed in a furnace oven (RhodeEcotop20, Helmut Rhode GmbH, Prutting, Germany), whereas the temperature is regulated via a custom-built-controller and tracked via an R Type thermocouple. This configuration guarantees a maximum deviation between setpoint and process temperature of ±2.0 °C for the heating rate, which is set to 3.0 °C/min with a dwell-temperature of 640 °C for 1 h. The decision to set 640 °C as the dwell-temperature was based on two main criteria. First, the annealing temperature needs to allow the PZT to form the perovskite phase and also avoid lead deficiency at the same time; i.e., a temperature between 550 and 640 °C is suitable [35]. Secondly, the temperature should allow parts of the TiO_2_ at the SiO_2_ interface to transform from the anatase phase into the rutile phase as this can affect the formation of perovskite PZT positively [30]. Although the exact temperature of this transition is vaguely defined, it occurs for pure TiO_2_ between 600 °C and 700 °C [36]. The cooling rate was limited to a maximum of 3.0 °C/min at first and then followed an exponential decay to avoid cracking. This temperature curve allows us to minimize cracks since the material PZT features in its perovskite structure a first order phase-transition at the material’s Curie temperature [35]. This fact mainly relates to the change in energy; i.e., the material adapts a tetragonal or rhombohedral instead of a cubic crystal lattice at the Curie temperature [30]. In addition to that, hillock formation due to bottom electrode’s materials can also contribute to the cracking of the piezoelectric layer, which is mainly associated with the expansion of the Pt layer due to the oxidation-diffused Ti [37]. Our applied target process temperature, and the assumed and corresponding phase or lattice changes according to Aungkavattana et al. are depicted in Figure 4 [38]. The semi-final step involves the patterning and deposition of the top electrode, for which 10 nm Ti and 170 nm Pt were sputtered once again (see Figure 3f).

The processed PMUT, or rather the piezoelectric layer, will feature a spontaneous polarization in six directions in the tetragonal phase (PbTiO_3_), and a spontaneous polarization in eight possible orientations for the trigonal phase [35]. It is for this reason that we polarize the PMUT as a final step by applying 15 V_DC_ for 20 min at room temperature, which will improve the PMUT’s deflection.

### 2.2. Material Characterization—Leakage Current and Polarization of the Piezoelectric Material

#### 2.2.1. Experimental Setup for the Material Characterization

**Figure 5:**
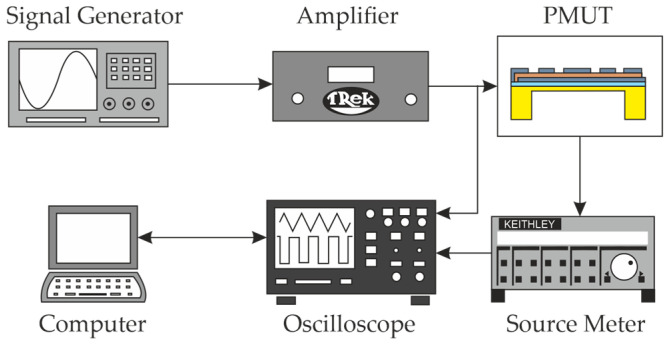
Experimental setup for measuring the hysteresis loops and I–V characteristics.

The leakage current and the PZT’s polarization determine the efficiency of the PMUT’s overall performance as a transducer. These two parameters are classified via a pulsed method as suggested in [35], for which a signal generator (Keysight Technologies, 33500B, Santa Rosa, CA, USA) provides a triangular signal. The signal itself is amplified (Advanced Energy Industries, Inc., TREK modelPZD700 M/S, Lockport, NY, USA) to account for the capacitive load of the PMUT, whereas the current of the load is monitored via a source meter (Keithley Instrument, 428 Source Meter, Solon, OH, USA). Both the current and the applied voltage to the PMUT are acquired via an oscilloscope (Keysight Technologies, DSOX6004A, Santa Rosa, CA, USA) as seen in Figure 5. The applied voltage V to the PMUT was defined as positive when the electric field E was oriented from one of the top electrodes to the bottom electrode.

**Figure 6:**
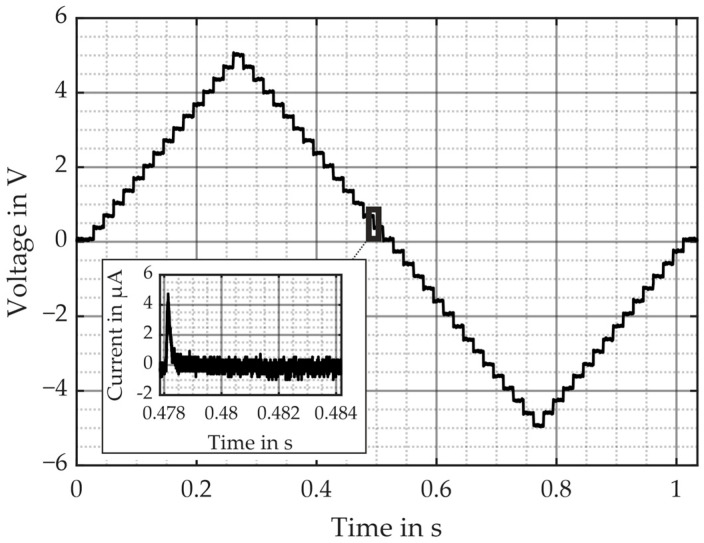
Applied voltage to the PMUT with Δt = 16 ms, and the mirrored and corresponding current profile for one particular voltage step with its current peak and current leakage.

This measurement method accounts for the current leakage $I_{leak}$ by introducing a hold time Δt for each particular voltage step as seen in Figure 6. At the beginning of Δt, the current will increase as the charge increases and will stabilize at a certain value at the end of Δt. This stabilized value defines the leakage current $I_{leak}$, which will be deducted from the measured current $I_{m}$ to yield the charge Q by

(3) $$Q=\int_{0}^{\Delta t}(I_{m}-I_{leak})\;dt,$$

which either charges or discharges the sample.

On the one hand, the selection of the hold time duration Δt and, thus, the overall frequency in which a voltage step is applied is influenced by the capacitive and resistive character of the PMUT and the resistance of the interconnections. This assumption implies that the change in charge will follow the characteristics of a regular resistor and capacitor (RC) in series circuit and can provide an estimate on the hold time Δt. The measured values by the LCR meter (Good Will Instrument Co., Ltd., LCR821, Taipei, Taiwan) of our probes, including the wires soldered directly on the samples’ contact pads, are roughly in the range of several 10 kΩ to 100 kΩ and 10^−9^ F. Considering the capacitance, and the measured resistive part, the time constant is approximately $\Delta t\approx R\cdot C\approx 100\;k\Omega \cdot 10^{-9}\;F\approx 100\;µs$.

On the other hand, a ferroelectric material’s change in polarization is defined by both a nucleation process, i.e., the formation of new domains, and a shift or growth of domain walls [41]. This fact also requires consideration as it may impact the charge flow and, thus, the choice of Δt. According to [35], one can assume the same mechanisms and time constants for perovskite PZT as they apply for BaTiO_3_. Whereas for low strength fields, the nucleation process is the dominating factor, the movement of the domain walls will dominate for higher field strength as the nucleation develops faster [41]. In case of perovskite PZT with a thickness of 1 µm, Δt≈ 1 µs already suffices to allow the nucleation process to be stabilized for each applied voltage level at room temperature [35].

In our particular case, the RC time constant and the nucleation process are approximately in the microsecond range, i.e., Δt yields 100 µs. In order to ensure that the charge indeed attains a constant value and the leakage current $I_{leak}$ is accurately determined over a longer period of time, we chose Δt=16 ms. The analysis of the current peak within the first 250 µs allows us to determine the charge according to Equation (3), whereas the current flow postponing the peak characterizes the leakage current $I_{leak}$. The estimation of $I_{leak}$ was obtained by calculating its average besides the peak. These settings result in a total of 60 steps, with a maximum and minimum voltage amplitude of ±5 V, respectively (see Figure 6).

The polarization *P* for each of these voltage levels is determined by

(4) $$P=\frac{Q}{A_{i}},$$

in which $A_{i}$ denotes the surface covered by each electrode ring i. The electric field *E* is defined by the applied voltage V and the PZT’s thickness d, i.e.,

(5) $$E=\frac{V}{d}.$$

#### 2.2.2. Results and Discussion

The advantage of this method is its capability to determine both the hysteresis loop at its operation points, and the I–V characteristics via the current leakage $\left|I_{leak}\right|$. The latter parameter accounts for the energy aspect in low-power devices, particularly in cases in which the leakage depends on the electrical connection.

**Figure 7:**
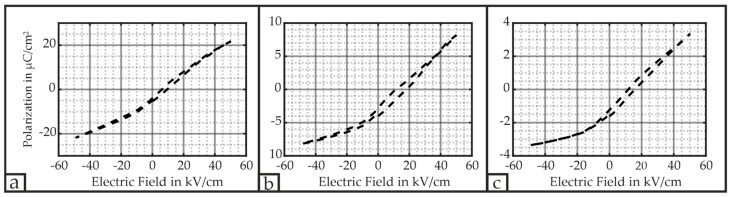
Respective P–E hysteresis loop of each ring—the loops rotate counterclockwise: (**a**) primary ring featuring a maximum of 21.8 µC/cm^2^; (**b**) 2nd electrode featuring a maximum of 8.2 µC/cm^2^; (**c**) 3rd electrode featuring a maximum of 3.4 µC/cm^2^.

Figure 7 depicts the P–E (polarization–electric field) hysteresis loop of the PMUT’s respective electrodes. In all three cases, the remanent polarization $P_{r}$ is approximately 0.7 µC/cm^2^, and the polarization at its peak values hardly shows clear saturation at the electric fields E of ±50 kV/cm. Therefore, the material’s property exhibits characteristics of hard PZT and has not been fully polarized. This aspect is particularly advantageous if the PMUT serves as an ultrasonic transmitter since the piezoelectric material returns to its initial state after the electric field is removed. Therefore, this hardly present memory effect allows for a reproducible behavior of the PMUT as an actuator and the deterministic emission of ultrasonic waves.

The maximum reached P decreases with an increasing ring number i; i.e., the primary electrode shows the highest value with 21.8 µC/cm^2^ at 50 kV/cm, whereas the 3rd electrode only reaches 3.4 µC/cm^2^ at the same electric field strength E. This fact implies that the sputtered PZT exhibits locally different behavior. We assume that the deposition process is the factor for this behavior. The materials Ti and Pt were not sputtered all over the wafer but only at the locations where the bottom electrode and the PZT layer were located. The PZT layer itself serves as an insulation layer; i.e., the material PZT covers the complete bottom electrode in order to prevent an electrical short circuit. According to [31], Ti-rich compositions tend to nucleate first. During the annealing process, the sputtered Ti diffuses through the bottom electrode. As the bottom electrode’s top and sides are exposed to PZT, the available Ti during the annealing process may not be sufficient to form high-quality PZT at these interfaces. This effect, which is localized at the position of the 3rd electrode, seems to be responsible for the measured values in Figure 7.

Despite these issues, the primary electrode’s 21.8 µC/cm^2^ at a field strength of 50 kV/cm is in the range as reported in [42] with approximately 27 µC/cm^2^ at 50 kV/cm, and is already higher than in [43], with approximately 6 µC/cm^2^ at 50 kV/cm. Additionally, these hysteresis loops only represent the minor loop corresponding to the voltage ranges, in which the PMUT will operate.

**Figure 8:**
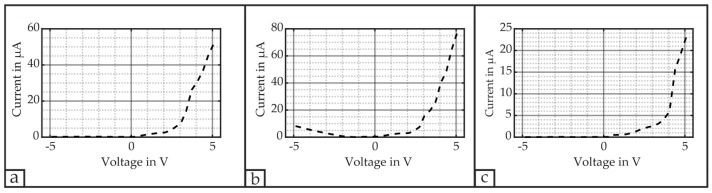
Current leakage |$I_{leak}$| of each respective electrode: (**a**) primary electrode drawing 0.21 µA at −5 V; (**b**) 2nd electrode drawing 8.4 µA at −5 V; (**c**) 3rd electrode drawing 0.01 µA at −5 V.

Figure 8 depicts the absolute leakage current |$I_{leak}$| for the applied voltage range of each respective electrode. The primary ring draws a maximum current of 52.2 µA at 5 V and 0.21 µA at −5 V, whereas the 2nd electrode draws 76.5 µA and 8.4 µA for the same voltage conditions. The 3rd electrode’s current consumption ranges between 23.0 µA and 0.01 µA. We assume micro cracks at the region of the second electrode to be responsible for an increase in the leakage current at the second electrode. However, the measured values are approximately in the range as reported in [44], in which a maximum current draw of approximately 22 µA at −3.1 V is stated, although a direct comparison between these values may be lacking due to a different PZT thicknesses of only 500 nm and different deposition conditions.

The primary and 3rd electrodes exhibit a non-linear I–V curve for which the drawn current increases exponentially at a certain positive voltage level with an increasing positive electric potential, while it remains close to zero for negative ones. Similar to the definition of the threshold voltage for diodes, we define the PZT’s threshold voltage $V_{TH-P}$ by extending the exponential curve to its intersection with the x-axis. In the case of the primary and 3rd electrodes, this $V_{TH-P}$ yields approximately 2.8 V and 3.5 V, respectively. The 2nd electrode features an increase in the current at both positive and negative voltage levels. Whereas the threshold voltage $V_{TH-P}$ = 2.9 V for the positive polarity, the threshold voltage cannot be determined for negative voltage levels as it does not follow an exponential characteristic but almost exhibits resistive features in this voltage range.

If the electrical connection of the positive and negative terminals is switched, the I–V curve will be symmetrically mirrored for all three electrodes at the y-axis corresponding to 0 V. Therefore, the connection of the electrical terminals would impact the overall energy demand if the PMUTs were driven as an embedded device.

The diode-like behavior of the V–I curves can relate to several factors such as the choice of the top electrode’s material, asymmetric electrodes or polarization [44,45]. Particularly, the PZT and Pt interface may be attributed to the formation of Schottky contacts [46]. However, in cases where the two electrodes are composed of the same material, only a small asymmetry should be present when different processing conditions apply [45]. Therefore, we assume that the polarization of the PMUT’s piezoelectric layer is the dominant factor as the current also increases exponentially for voltage levels applied opposite to the material’s polarization. In this context of polarization, it is the internal electric field of the piezoelectric layer that contributes to the asymmetry as it lowers or increases the potential barriers at the film interfaces dependent on the applied voltage [44].

## 3. Displacement Dynamics

### 3.1. Experimental Setup for the Displacement Dynamics

**Figure 9:**
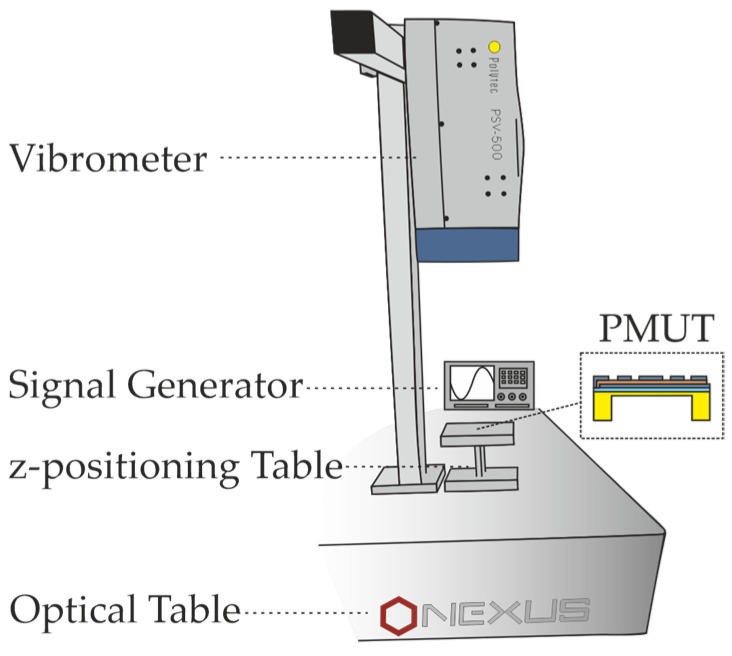
Experimental setup for the examination of the displacement dynamics.

The experimental setup comprises a laser Doppler vibrometer (Polytec GmbH, PSV-500, Waldbronn, Germany) placed on an optical table (Thorlabs, Inc., Nexus Optical Table, Newton, NJ, USA) that allows us to dampen any mechanical vibrations, which may impact the PMUT’s displacement. The installed micro lenses (Polytec GmbH, OFV-CL80, Waldbronn, Germany) improve the laser spot diameter to 7 µm. Each point of the PMUT’s surface is measured for 10 periods of the set frequencies, and then, the average deflection is calculated. The PMUT itself is additionally mounted on a z-positioning table enabling the PMUT to be focused on. The probe is directly excited via a two-channel signal generator (Keysight Technologies, 33500B, Santa Rosa, CA, USA) by a sinusoidal signal with an amplitude of 500 mV. The entire experimental setup is also shown in Figure 9.

Depending on the perspective, the displacement is normalized to 1 V, which is either the amplitude of the set voltage at the signal generator, or the actual measured voltage at the sample. The first approach focuses on the PMUT’s feature to be driven directly by a regular 50 Ohm output. The second approach accounts for the capacitive and resistive character of the PMUT, which yields higher voltages at the sample than actually set at the signal generator. In the following, we introduce two definitions which will allow us to differentiate the normalized displacement in relation to the applied voltage. The first approach will be referred to as VSAG (voltage set at generator), whereas the second approach will be denoted as AMV (actual measured voltage).

There are different excitation possibilities which are investigated as a proof of concept, all of them focusing on the (0,1)-mode. These possibilities include the following:

- The excitation of all three electrodes simultaneously.
- Separate excitation of each electrode.
- Excitation of the primary electrode with the resonance frequency and applying different DC voltages at the 3rd electrode.
- A new concept which we will refer to as Ring Impinging (RI). The primary electrode and the 3rd electrode will each be excited with a different frequency still within their bandwidth *B*.

### 3.2. Simultaneous Excitation of All Three Electrodes and Separate Excitation of Each Electrode

For the single excitation of each separate electrode, the non-driven electrodes are shortened; i.e., the electrode is connected to the ground signal. Additionally, the quality factor $Q_{B}$ is determined via

(6) $$Q_{B}=\frac{f_{0}}{B},$$

where $f_{0}$ denotes the resonance frequency and B the 3 dB bandwidth. Both values are determined as either the maximum deflection or the deflection reduced to 70.7% of its maximum value, respectively.

**Figure 10:**
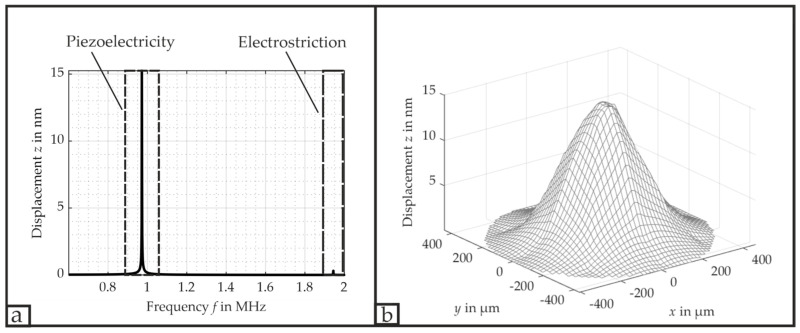
Displacement dynamics of the PMUT. (**a**) FFT spectrum of the PMUT’s center with an excitation frequency $f_{0}$ = 971.7 kHz; (**b**) measured deflection of the PMUT’s (0,1)-mode via the laser Doppler vibrometer.

Figure 10a shows the acquired FFT (Fast Fourier Transform) spectrum of the PMUT’s center when it is excited with all three electrodes at its resonance frequency. In this spectrum, the deflection caused by electrostriction is only slightly present at twice the resonance frequency $f_{0}$ with 0.3 nm; i.e., the piezoelectric effect remains the primary factor for the PMUT’s deformation.

Figure 10b depicts the corresponding PMUT’s (0,1)-deflection at its resonance frequency $f_{0}$ = 971.7 kHz when all three electrodes are excited simultaneously. The sinusoidal amplitude of 500 mV at the signal generator resulted in a deflection of 15.20 nm, leading to a VSAG = 30.50 $\frac{nm}{V}$. As the voltage drop across the PMUT itself corresponded to 890 mV, the AMV = 17.13 $\frac{nm}{V}$. The bandwidth *B* is approximately 15.6 kHz, resulting in a $Q_{B}=62.2$.

**Figure 11:**
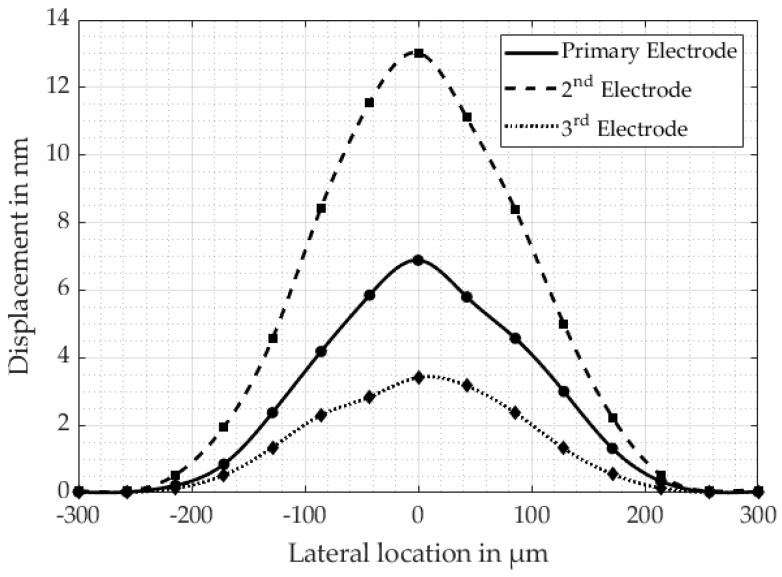
Measured PMUT deflections in the (0,1)-mode of each electrode being individually excited with 890 mV. The radii of the electrodes exhibit $r_{Primary}$ = 100 µm, $r_{2nd}$ = 190 µm, $r_{3rd}$ = 280 µm, respectively.

Figure 11 provides further information on the different (0,1)-mode deflections at the resonance frequencies of each separately driven electrode and an applied voltage of 890 mV, whereas a slight asymmetry in the deflection is noticeable. For this configuration, we did not detect any significant phase differences between the driving electrode and the shorted electrodes. Moreover, Table 1 lists the complete measured resonance frequencies, displacements, bandwidths B and quality factors $Q_{B}$ for the different electrical connections of either all three electrodes connected simultaneously or each electrode separately. The resonance frequency $f_{0}$ remains approximately the same for all connections; only when the 3rd electrode is solely connected does the configuration exhibit a slight shift of approximately 2 kHz towards $f_{0}=973.6$ kHz. This minor shift is probably introduced due to some minor differences at the electrical connection to the PMUT and the clamping at the membrane’s edges.

According to Table 1, the bandwidth B has the greatest value of 15.6 kHz for all three electrodes being excited simultaneously, remains approximately the same for the primary electrode and 3rd electrode at around 12 kHz, and decreases to its minimum of 9.8 kHz for exciting only the 2nd electrode. As a consequence, $Q_{B}$ increases respectively. We assume the reason for this behavior to be a combination of passive mass and boundary conditions. In cases for which the PMUT exhibits a reduced deflection at the single electrode excitation, the absolute difference between the maximum deflection and the 3 dB value decreases at the same time and, thus, yields a higher bandwidth B in a relative perspective. In cases for which the PMUT is driven by all three electrodes, the slight resonance shift of the 3rd electrode seems to contribute to a greater bandwidth B.

The PMUT shows the greatest displacement when all three electrodes are excited simultaneously, whereas for a single electrode’s excitation, the 2nd electrode deforms the membrane the most with a VSAG = 26.04 $\frac{nm}{V}$, and the 3rd electrode the least with a VSAG = 6.82 $\frac{nm}{V}$. The reason for its different deflection is attributed to the differences in the electrode’s area covering the PZT layer, the boundary conditions, passive load and the piezoelectric characteristics of the PZT at its respective location. In order to evaluate these pieces of information, we introduce a new quality parameter G, which will take into account the maximum polarization P and the covered area of the individual electrode A in relation to the measured maximum deflection VSAG according to

(7) $$G=\frac{VSAG}{P\cdot A}.$$

Table 2 lists each parameter of the individual electrodes and the corresponding quality parameter G. The best properties are attributed to the 2nd electrode, followed by the primary and then 3rd electrode. In the case of the single excitation of either the primary electrode or the 2nd electrode, the dominating factor for the 2nd electrode’s superior deflection is the covered area A, since the piezoelectric properties are superior for the primary electrode. Considering only the 3rd electrode, the footprint of this electrode covers almost four times the area of the primary electrode but generates the smallest deflection. Thus, the observation must be attributed to the piezoelectric characteristics as the primary cause, and also the membrane’s clamping.

Despite the different maximum deflections, the PMUT’s feature to be driven by either electrode adds redundancy to the overall system, which may be required for implantable devices as a safety element. Additionally, the emitted pressure can be adjusted without the need to modify the applied voltage at the transducer itself.

### 3.3. Deflection Adjustment via the 3rd Electrode

The experiment intends to be an initial evaluation for the deflection adjustment of the PMUT via the 3rd electrode, whereas an adjustment via the 2nd electrode will not be investigated due to its leakage current of up to 76.5 µA. For this experimental setup, the primary electrode is excited with its resonance frequency $f_{0}$ = 971.7 kHz, and the 3rd electrode with a ±5 V_DC_ potential.

By applying a 5 V_DC_ potential at the electrode, the membrane’s deflection can be adjusted by a factor of 2.8. In this case, the piezoelectric layer either softens in its forward direction or hardens for the reverse bias. As a result, the deflection increases for a softened membrane to a VSAG = 14.2 $\frac{nm}{V}$, while a harder PZT layer reduces it to a VSAG = 5.34 $\frac{nm}{V}$, provided the frequency remains the same in both cases. We also assume the behavior to be non-linear due to the diode-like behavior as described in the section of the material’s characterization.

### 3.4. Ring Impinging (RI)

This configuration involves the primary electrode’s excitation with its resonance frequency $f_{0}$ = 971.7 kHz, and the 3rd electrode’s excitation with a different frequency $f_{M}$ = 965.8 kHz, which is still within its bandwidth B. The idea is to keep the PMUT’s deflection at its (0,1)-mode for efficient emission of ultrasonic waves while emitting two distinguishable frequencies simultaneously. Therefore, the choice of these two frequencies is based on the third electrode’s capability to still deflect the membrane, whereas the laser Doppler vibrometer’s frequency resolution should allow for the detection of a different frequency. The emission of two ultrasonic waves is particularly advantageous for ultrasonic-based communication as it allows us to implement frequency mixing.

**Figure 12:**
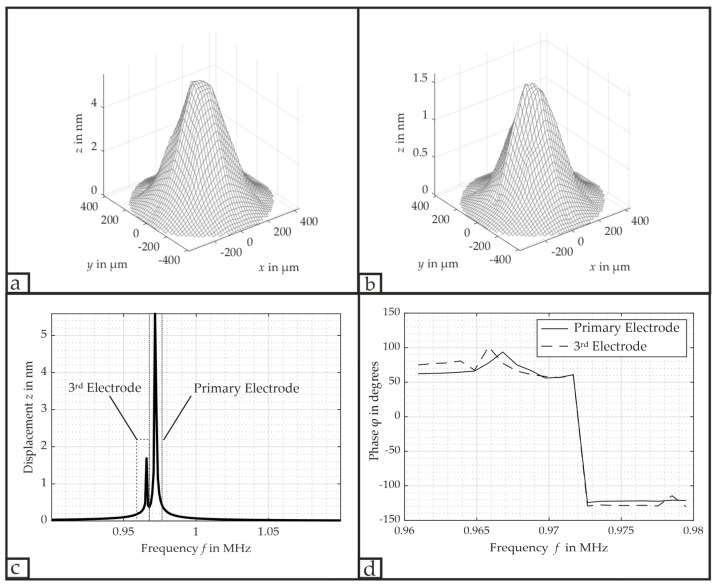
Measured displacement dynamics and frequency spectrum of the PMUT. (**a**) (0,1)-mode displacement due to the primary electrode response with $f_{0}$ = 971.7 kHz; (**b**) (0,1)-mode displacement due to the 3rd electrode’s response with $f_{M}$ = 965.8 kHz; (**c**) FFT spectrum acquired via the vibrometer at the PMUT’s center, confirming the presence of both $f_{0}$ = 971.7 kHz (primary electrode) and $f_{M}$ = 965.8 kHz (3rd electrode); (**d**) measured phase *φ* of the primary electrode and the 3rd electrode.

Figure 12 shows both the measured deflection of each frequency and the FFT acquired via the laser Doppler vibrometer at the center of the PMUT, along with the measured phases *φ* of the primary and 3rd electrode. The deflection of the primary and 3rd electrode yielded a maximum VSAG with 11.17 $\frac{nm}{V}$ and 3.37 $\frac{nm}{V}$, respectively. There also exists no noticeable phase difference between the primary electrode and the 3rd electrode; i.e., the excitation results in constructive interference. As both frequencies can be emitted simultaneously in the (0,1)-mode, the acoustic pressure field will contain both frequencies. Additionally, these two frequencies will also superimpose over time, resulting in a change in the PMUT’s amplitude deflection $A_{res,m}$ according to

(8) $$A_{res,m}=A_{1,m}\cdot \sin\left(2\pi \cdot f_{0}\cdot t\right)+A_{2,m}\cdot \sin(2\pi \cdot f_{M}\cdot t),$$

where $A_{1,m}$ denotes the deflection due to $f_{0}$, and $A_{2,i}$ denotes the deflection due to $f_{M}$, each at their respective location.

**Figure 13:**
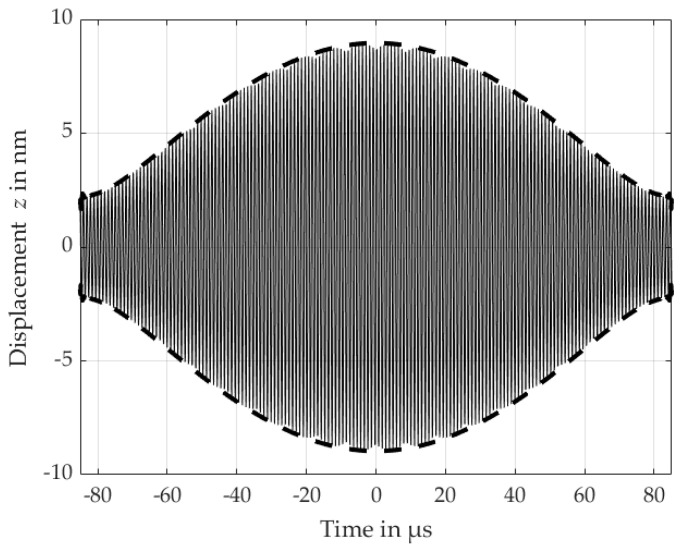
Estimated superposition of $f_{0}$ = 971.7 kHz and $f_{M}$ = 965.8 kHz with their respective amplitudes $A_{1}$ = 5.59 nm and $A_{2}$ = 3.37 nm at the PMUT’s center over time, yielding an envelope with a frequency $f_{e}$ = 5.9 kHz.

This superposition results in an additional amplitude-modulation, which is detectable via its envelope, as seen in Figure 13. The envelope’s frequency $f_{e}$ = 5.9 kHz yields the difference between the two excitation frequencies $f_{0}$ and $f_{M}$, and is also referred to as the beat frequency. As the envelope itself is a deterministic representation of the superposition, it allows us to retrieve the two underlying frequencies and, therefore, may require less hardware-intensive-detection designs. Additionally, the beat frequency may be beneficial in cases where the two higher frequencies are too attenuated due to the propagation medium. In this case, the medium functions as a lowpass filter due to frequency dependent attenuation, through which the beat frequency may still propagate.

## 4. Summary, Conclusions and Outlook

In this contribution, a ring-structured PMUT was fabricated and investigated which featured the ability to individually excite each of the three electrode rings. The piezoelectric material’s properties were determined via triangular signal, resulting in a maximum polarization P of 21.8 µC/cm^2^, 8.2 µC/cm^2^ and 3.4 µC/cm^2^ at their respective electrode location under a maximum electric field of ±50 kV/cm. These piezoelectric material characteristics also exhibited diode-like behavior; i.e., it shows a forward and reverse bias behavior. The minimum current at their relatively maximum excitation voltage of −5 V yields 0.21 µA for the primary electrode, 8.4 µA for the second electrode and 3.4 µA for the third electrode.

Additionally, the displacement dynamics of the PMUT were analyzed for which all electrodes were excited either simultaneously or each electrode individually. The maximum displacement yielded 30.50 nm per applied volt at the signal generator when all three electrodes are excited simultaneously with the same frequency and phase. Moreover, an individual electrode actuation also allowed the PMUT to deflect in its (0,1)-mode with a reduced displacement. This feature adds redundancy in terms of a safety feature in cases where the contact terminals or the PZT deteriorate at different locations during the lifecycle of the device, as it ensures a stable emission or detection of ultrasonic waves. This aspect is also beneficial for deeply seated implantable devices, for which ultrasound can serve as a reliable communication link. At the same time, it also provides the possibility of pressure adjustment without the need for additional hardware that regulates the voltage and, therefore, the related pressure. This holds particularly true if the PMUT were to be driven directly by a digital logic.

Besides a single excitation of the electrodes, a DC potential also affects the PMUT’s deflection. For this configuration, the primary electrode was excited, and the third electrode is biased via a DC potential. Due to the softening or hardening of the piezoelectric material PZT, the deflection can be adjusted by a factor of 2.8.

The separate excitation of electrodes also enables new possibilities in terms of ultrasonic-based communication as two different distinguishable frequencies can be emitted simultaneously, which allows us to implement frequency mixing. In our case, the third electrode induced a (0,1)-mode displacement of 3.37 nm with a frequency of 965.8 kHz, and the primary electrode a (0,1)-mode displacement with 11.17 nm with a frequency of 971.7 kHz. As both deflections will superimpose over time, this results in a deterministic envelope exhibiting a reduced frequency $f_{e}$ = 5.9 kHz. This frequency can be detected at lower operating frequencies, i.e., detection in the baseband, and thus reduce hardware demands at the receiver’s side.

Future work will focus on several diameters with a ring-structured electrode design, the manufacturing of an array and the characterization of the actual respective pressure fields in water. Such work may also include a detailed investigation into the change in deflection for several DC voltages or the course of differently introduced phase shifts.

## Figures and Tables

**Table 1:** Resonance frequencies $f_{0}$, displacements, bandwidths B and quality factors $Q_{B}$ for various electrical connections.

Excited Electrodes	$f_{0}$ in kHz	B in kHz	$Q_{B}$	VSAG in $\frac{nm}{V}$	AMV in $\frac{nm}{V}$
All three electrodes	971.7	15.6	62.2	30.50	17.13
Primary Electrode	971.7	11.7	83.1	13.78	7.74
2nd Electrode	971.7	9.8	99.1	26.04	14.63
3rd Electrode	973.6	12.7	76.7	6.82	3.83

**Table 2:** Maximum polarization P, displacement VSAG, covered area A by the electrode and quality factor G for various electrical connections.

Electrode	P in $\frac{µC}{cm^{2}}$	VSAG in $\frac{nm}{V}$	A in $cm^{2}$	G in $\frac{nm}{V\cdot µC}$
Primary Electrode	21.8	13.78	3.14 · 10^−4^	2013
2nd Electrode	8.2	26.04	6.8 · 10^−4^	4670
3rd Electrode	3.4	6.82	1.2 · 10^−3^	1672

## Data Availability

The original contributions presented in the study are included in the article, further inquiries can be directed to the corresponding author.

## Abbreviations

The following abbreviations are used in this manuscript:

$A_{i}$	Area
$A_{res,m}$	Amplitude
$A_{1,m},A_{2,m}$	Amplitude
AIN	Aluminum Nitride
AMV	Actual Measured Voltage
α	Nondimensional Parameter
B	Bandwidth
BaTiO_3_	Bariumtitanate
C	Capacitance
CMOS	Complementary metal-oxide semiconductor
CMUT	Capacitive Micromachined Ultrasonic Transducer
D	Flexural Rigidity
d	Thickness
DSE	Deep Silicon Etching
E	Electric Field
FFT	Fast Fourier Transform
f	Frequency
$I_{m},I_{leak}$	Current
$J_{n}(\mu )$	Bessel Function
j	Imaginary Unit
LDV	Laser Doppler Vibrometer
O	Oxygen
P	Polarization
PMUT	Piezoelectric Micromachined Ultrasonic Transducer
PZT	Lead Zirconate Titanate
Pt	Platinum
Q	Charge
φ	Radiation angle, phase
R	Resistance
r	Radius
RI	Ring Impinging
s	Stiffness
Si	Silicon
SiO_2_	Silicon Dioxide
t	Time
T	Tension
Ti	Titanium
TiO_2_	Titanium Dioxide
V	Voltage
VSAG	Voltage Set at Generator
$Z_{0},\;Z$	Acoustic Impedance
Z_n_O	Zinc Oxide
